# The First Outbreak of Eastern Equine Encephalitis in Vermont: Outbreak Description and Phylogenetic Relationships of the Virus Isolate

**DOI:** 10.1371/journal.pone.0128712

**Published:** 2015-06-04

**Authors:** Kali D. Saxton-Shaw, Jeremy P. Ledermann, Joan L. Kenney, Erica Berl, Alan C. Graham, Joel M. Russo, Ann M. Powers, John-Paul Mutebi

**Affiliations:** 1 Centers for Disease Control and Prevention, Division of Vector Borne Infectious Diseases, Fort Collins, Colorado, United States of America; 2 Vermont Department of Health, Burlington, Vermont, United States of America; 3 Vermont Agency of Agriculture, Food & Markets, Waterbury, Vermont, United States of America; 4 United States Department of Agriculture, APHIS VS, Montpelier, Vermont, United States of America; University of California Davis, UNITED STATES

## Abstract

The first known outbreak of eastern equine encephalitis (EEE) in Vermont occurred on an emu farm in Rutland County in 2011. The first isolation of EEE virus (EEEV) in Vermont (VT11) was during this outbreak. Phylogenetic analysis revealed that VT11 was most closely related to FL01, a strain from Florida isolated in 2001, which is both geographically and temporally distinct from VT11. EEEV RNA was not detected in any of the 3,905 mosquito specimens tested, and the specific vectors associated with this outbreak are undetermined.

## Introduction

The first isolates of Eastern equine encephalitis virus (EEEV) were obtained from the brains of encephalitic horses during an epizootic in Delaware, Maryland, Virginia and New Jersey in 1933 [1]. In 1938 EEEV was isolated from the brains of several fatal pediatric encephalitis cases and it was implicated as the causative agent [2,3]. Subsequent ecological and laboratory studies indicated that EEEV is primarily maintained in enzootic cycles in which the virus is transmitted among susceptible birds, especially passerine birds, by the ornithophilic mosquito *Culiseta melanura* [4–9]. Epizootic and epidemic transmission occurs only intermittently, typically during seasons of high vector population density, which are usually preceded by periods of above normal precipitation [10]. During spillover transmission, atypical hosts such as horses and humans become infected with EEEV and while these hosts are highly susceptible to EEEV, ecologically they are dead-end hosts as neither generate enough viremia to infect mosquitoes and perpetuate the transmission cycle [11]. Spillover events may include transmission by *Cs*. *melanura*, *Coquillettidia perturbans*, *Aedes canadensis*, *Ae*. *sollicitans*, *Ae*. *taeniorhynchus* and several other species [7–8,11–15]. The specific overwintering mechanism or mechanisms are currently not well understood although there is evidence of both local persistence as well as re-introduction of new strains from warmer climates [8,15].

Since 2005, there has been increased detection of EEEV activity in northeastern US [16–18] with activity reported in New Hampshire [19], Massachusetts [18], Maine [20], New York [16], Connecticut [19] and recently in Vermont [21,22]. The EEEV activity in Vermont is particularly intriguing because despite well documented EEEV activity in all the surrounding states and territories [8,15–19,23], there was no documented detection of EEEV activity in Vermont until the fall of 2010 [21,22]. In this manuscript we describe the first outbreak of EEE in Vermont and the phylogenetic relationships of the first EEEV isolate from this state.

## Materials and Methods

### Sample collection and Virus isolation

**Fig 1:**
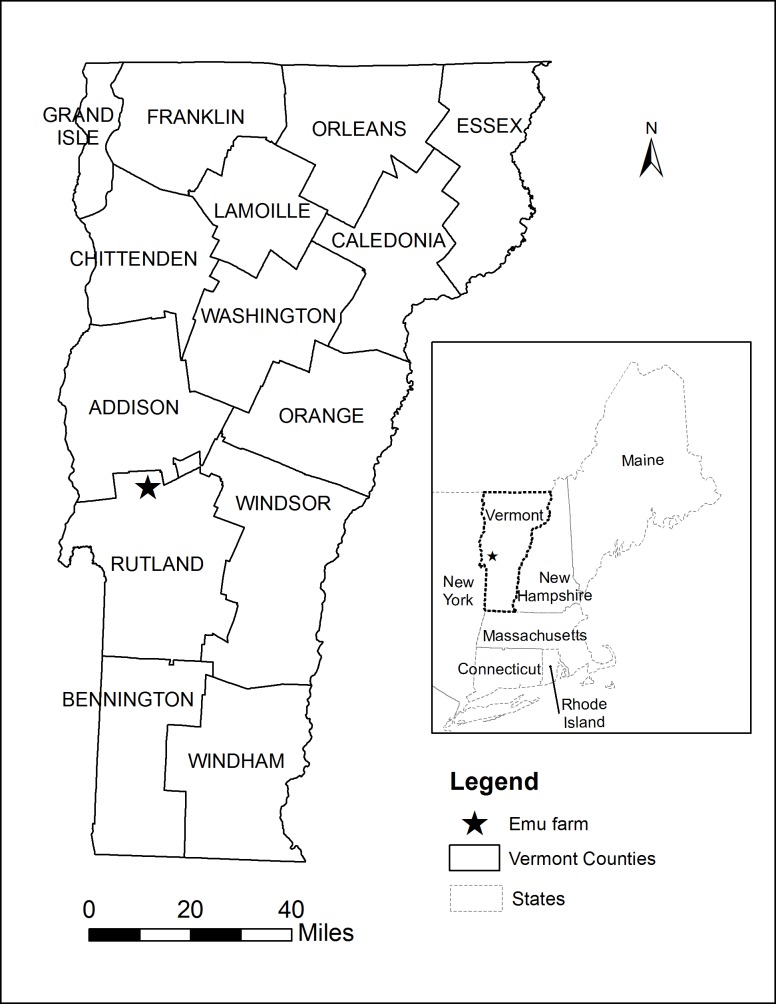
A map of Vermont showing the location of the emu farm in Rutland County where the EEE outbreak occurred in 2011.

On September 21^st^, 2011, a local veterinarian performed a necropsy on one of the dead emus from a farm in Rutland County (Fig 1). Brain tissue was collected and sent to the New Hampshire Department of Health’s Public Health Laboratory for PCR testing for the presence of EEEV RNA. On September 22^nd^, 2011, the lab confirmed that the PCR test was positive for EEEV. The sample was then sent to the Centers for Disease Control in Fort Collins, CO for additional characterization.

Upon arrival at the CDC laboratory in Fort Collins, CO, the virus, *Rutland_Co_VT2011_Emu* (VT11, Genbank Accession number: JX2623861), was isolated from the brain homogenate through a single passage in Vero cells. Viral RNA was extracted using the QIAamp viral RNA protocol (Qiagen, Valencia, CA). The OneStep RT-PCR protocol (Qiagen, Valencia, CA) was utilized to generate PCR amplicons for classical sequencing using virus-specific primers (Table 1) designed within the Primer Select program (DNASTAR, Madison, WI). Appropriate amplicons were cleaned using the MinElute Gel Extraction Kit (Qiagen, Valencia, CA). The DNA was subsequently sequenced using virus-specific primers and the Big Dye v3.1 kit on an ABI 3130xl genetic analyzer (Applied Biosystems, Foster City, CA). The VT11 sequence was assembled and analyzed for sequence quality and genome coverage using Lasergene suite software (DNASTAR, Madison, WI).

**Table 1:** The oligonucleotides used for EEEV full genome sequencing.

Name	Use	Sequence	Tm (C)
EEE 40 FWD	RT-PCR and sequencing	GGCAACCACCCTATTTCCACCTA	70
EEE 626 FWD	Sequencing	GGCGCCTACCCTACATACA	60
EEE 1087 FWD	Sequencing	ATGACCGGGATACTGGCGACTGAC	76
EEE 1349 REV	Sequencing	GGCGGGCACCTTCTTAATAGTTTG	72
EEE 2059 REV	RT-PCR and sequencing	GCATCCCCTTTCTTCACGCACTTC	74
EEE 1824 FWD	RT-PCR and sequencing	CGCGCAGGACGATACAA	54
EEE 2586 FWD	Sequencing	CTCGGCGATGCACTAAGAC	60
EEE 3030 FWD	Sequencing	CATGGCGAAAATACTTGAGAC	60
EEE 3438 FWD	Sequencing	ATAACCCGCTAATAAATGT	50
EEE 4035 REV	RT-PCR and sequencing	TTATGTCGCCGCGCACCACTCTAT	74
EEE 3836 FWD	RT-PCR and sequencing	GCAGTCGCCCGCTCATTCA	62
EEE 4619 FWD	Sequencing	CCGAGGGCAAGGTGTATT	56
EEE 4956 REV	Sequencing	GCCGGGGGTACAGTGCCAGAGA	74
EEE 5102 FWD	Sequencing	TGCAAGAATCCCCAGCCCTCCAT	72
EEE 5742 REV	RT-PCR and sequencing	GTTTGCATTGCCGCGTAGATTTTT	68
EEE 5568 FWD	RT-PCR and sequencing	GAGCCGCAGCGCAGACACGAT	70
EEE 6252 FWD	Sequencing	ATTTGCGGCCTGAGATACG	58
EEE 6562 REV	Sequencing	ACATCCCGTTTAAGGTCCATC	62
EEE 7081 FWD	Sequencing	CGCAGCATTTATCGGCGACGACAA	74
EEE 7776 REV	RT-PCR and sequencing	CGTTTGGCGGGCGGTCCTG	66
EEE 7509 FWD	RT-PCR and sequencing	CCATAACCCTCTACGGCTGACCTAAAT	65
EEE 8352 FWD	Sequencing	AAAGGGGGTTACAGTCAAAGATAC	68
EEE 8666 REV	Sequencing	GCATGCGCATCCCCTCTGACTTC	74
EEE 9403 REV	RT-PCR and sequencing	TGGTCCGGGTGCAGGTGTAAAATC	74
EEE 9153 FWD	RT-PCR and sequencing	CGAGCGGCGCCCAAGTGAAATA	70
EEE 9536 FWD	Sequencing	CCGGAGAAGGGTTGGAGT	58
EEE 9877 REV	Sequencing	GGATAAGCGTCTGCATCCAG	64
EEE 10466 FWD	Sequencing	ATGGTGAAACTCCCGCGAAAATAG	70
EEE 11183 REV	Sequencing	TCGCCGACGTAAAGGATTC	58
T25 REV	RT-PCR and sequencing	T(25)V	78

### Phylogenetic analysis

**Fig 2:**
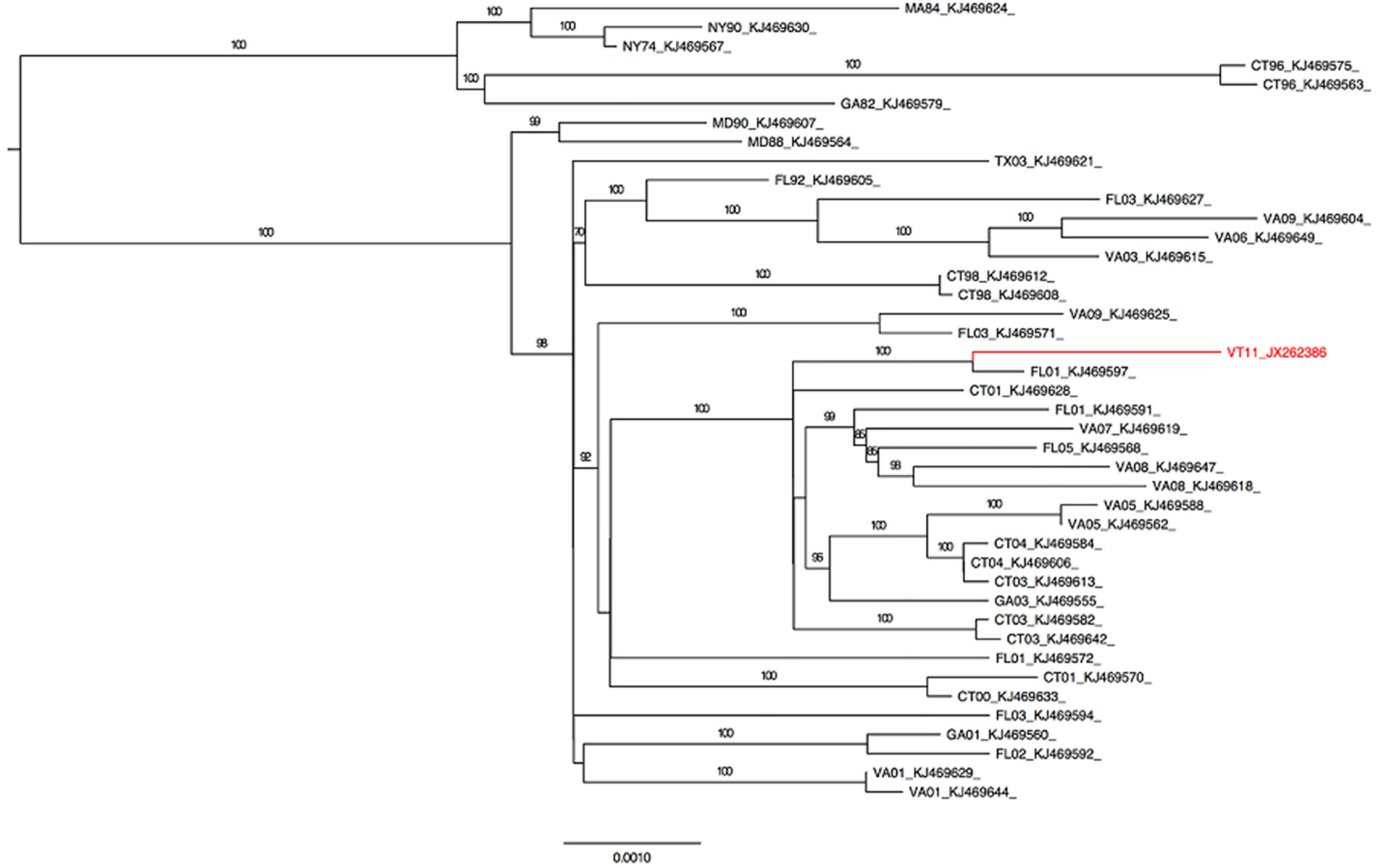
A maximum likelihood tree for 42 full genome sequences of EEEV isolates isolated from eastern US. VT11 is highlighted in red print. Numbers at nodes are bootstrap values based on 1000 replicates.

Since there were no other sequences of EEEV from Vermont, studies were undertaken to determine genetic relationships with other strains from the US. Sequences were selected from GenBank (Fig 2) to represent isolates from diverse counties in multiple US states. Sequences for the E2 glycoprotein gene and the full coding sequences were aligned using MUSCLE on the Cipres Science Gateway [24,25]. Maximum likelihood inference was performed using RAxML 7.06 on the Cipres Science Gateway [26]. 1000 replicates of bootstrapping resampling were utilized to assess the accuracy of tree topologies. Output trees were generated for publication using FigTree v1.4.

### Mosquito collection and processing

The Vermont Department of Health (VDH) and the Vermont Agency of Agriculture, Food and Markets (VAAFM) routinely conduct statewide mosquito based surveillance at 77 trap sites. They monitor CDC gravid traps, resting traps and CDC CO_2_-baited light traps at all the sites. Mosquitoes were collected once a week, beginning in mid-June and ending in mid-October, and identified to species by using the keys of Darsie and Ward [27]. Mosquitoes of the same species and sex were pooled into groups of 50 or less and screened for EEEV antigen using the VecTest (Medical Analysis Systems, Inc., Camarillo, CA). During the outbreak, additional traps were placed at several locations close to the emu farm to capture mosquitoes at the outbreak site with the aim of incriminating the vector(s) associated with the outbreak.

## Results

### Outbreak description

On September 21, 2011, the VDH was notified by the Assistant State Veterinarian at the VAAFM about an emu flock in Rutland County (Fig 1) that had suffered multiple fatalities from illness starting on September 15^th^. By September 21^st^, 14 emus had died with symptoms of hemorrhagic gastroenteritis. Additional emus showed symptoms of illness, including bloody discharges, weakness and inability to stand. In total, 19 emus died over a 10 day period. All ages were affected: 3 of 44 emus less than 6 months old, 9 of 27 emus 16–18 months old, and 7 of 22 breeder stock birds which were over 4 years old. Two additional birds became ill but survived resulting in an overall attack rate of 22.6%.

### Phylogenetic analysis

Despite the limited availability of full-length coding sequences from recent isolates, phylogenetic analyses using sequences from the range of North American strains revealed that VT11 has the highest identity with an isolate from Florida in 2001 (FL01 KJ469597) with a pairwise identity of 99.8% across the coding regions (Fig 2). In all analyses performed (using the range of sequences available), FL01 was the only strain that shared a common ancestor with VT11. However, VT11 also showed high similarity (6 or fewer nucleotide differences) to other viruses within the observable polytomy including strains from Connecticut, New Hampshire, Virginia, Florida, and Georgia [CT01 (KJ468628), NH05 (KJ469631 and KJ469556), VA08 (KJ469647 and KJ469618), FL05 (KJ469568), CT03 (KJ469642 and KJ469613), VA05 (KJ469588), FL01 (KJ469591), VA07 (KJ469619), and GA03 (KJ469555)]. Given the low degree of sequence variation, all analyses had low bootstrap values and poor basal resolution, as expected.

### Mosquito surveillance

In 2011, 42,129 mosquitoes belonging to 8 genera and 30 species were captured between June and October. Of these, 3,905 (9.27%) (Table 2) were screened for EEEV antigen. No positive results were obtained, which was possibly due to the small test sample size or the lack of sensitivity of the assay. However, the mosquito trapping did indicate a strong presence of species previously associated with EEEV transmission in North America. For example, there were 1,070 *Cs*. *melanura* [8], 7,758 *Cq*. *perturbans* [8], 429 *Ae*. *canadensis* [8], 17,291 *Aedes vexans* [8], and 279 *Culiseta morsitans*) [8] which comprised 2.54%, 18.42%, 1.02%, 41.08% and 0.66% of the total collections, respectively. Although EEEV antigen was not detected in any of the 2011 specimens tested, these species are capable of both epizootic and epidemic transmission of EEEV [8,11–15]. In subsequent years in Vermont, a total of 40 EEEV positive mosquito pools were detected from *Cs*. *melanura*, *Cq*. *perturbans*, *Cs*. *morsitans* and *Ae*. *canadensis* (Table 1) which were abundant species during the 2011 outbreak and were the most likely vectors for that outbreak.

**Table 2:** The number and percentage of mosquitoes collected and screened for EEEV in Vermont from 2011 to 2014.

Year	Number of Mosquitoes Collected	Number of Mosquitoes Tested	EEEV Positive Pools	EEEV Positive Species**
2011*	42,129	3,905 (9.27%)	0	
2012	10,498	4,676 (44.27%)	10	*Cs*. *melanura* (10)
2013	32,727	16,729 (51.12%)	22	*Cs*. *melanura* (20)
				*Cq*. *perturbans* (1)
				*Cx*. *pipiens/restuans* (1)
2014	67,335	41,700 (61.93%)	8	*Cs*. *melanura* (5)
				*Cs*. *morsitans* (2)
				*Ae*. *canadensis* (1)

* Tested using the VecTest. 2012–2014 samples were tested using RT-PCR (27)

** Number in parenthesis indicates number of positive pools.

## Discussion

The first known outbreak of EEE in Vermont occurred on an emu farm in Rutland County in September 2011. The outbreak was restricted to emus on only one farm and no other farm animals, domestic animals, or humans were infected. The outbreak was intriguing because there were no detections of EEEV activity in the state before 2010 [21,22] yet EEEV activity had been previously detected in all surrounding states and territories including Quebec, Canada, directly north of Vermont [8,15–21,28]. The detection of EEEV antibodies in free-ranging deer and moose in Vermont in 2010 [21,22] in concert with the detection of human infections in 2012 and detection of EEEV RNA in field-collected mosquitoes in 2012, 2013 and 2014 (Table 1) leads to at least two possibilities for EEEV presence in the state. Either EEEV was only recently introduced into Vermont and the period from 2010 to 2014 represents the first major emergence events, or EEEV had been circulating enzootically in Vermont but was simply undetected until 2010.

Based on phylogenetic analysis (Fig 2) and pairwise sequence differences, VT11 is most closely related to a strain from Florida in 2001 (KJ469597), which is both geographically and temporally distinct from VT11. The isolate was also found to be highly similar to other virus strains from northeastern US including those from Connecticut (2001–2004) and Virginia (2005–2008) (Fig 2). It is difficult to draw conclusions regarding the importance of geographically similar viruses due to the general lack of temporal overlap; however the observed clustering is consistent with what is known about North American EEEV ecology. Maintenance by avian hosts that allows for wide geographic distribution and the introduction of genotypes from remote geographical locations, particularly along seasonal flyway patterns, contributes to limited genetic diversity. This is a likely explanation as to why the VT11 sequence has such a high degree of homology with a geographically disparate isolate such as FL01. However, examples of regionally confined evolution of EEEV with occasional introductions from geographically distant locations has been observed multiple times [19,23,29–31], and there is much evidence to support the presence of and continued spread of enzootic EEEV in the North eastern states [19–23]. So while it is probable that this particular epizootic was engendered from a novel introduction from Florida, the potential for future epizootic spillover from the recently identified enzootic foci in Vermont deer and moose is also a concern [21,22].

Studies of *in vivo* infection of emus with EEEV, have shown that they develop a high-titered viremia [greater than 9.0 log_10_ (PFU/ml)], and shed virus in secretions and excretions increasing the risk of transmission to adjacent animals and human caretakers [32]. Such a high-titered viremia could have increased the likelihood of virus acquisition and transmission by resident vector populations, especially less competent mosquitoes or atypical vectors, which could have potentially acted as bridge vectors.

These finding highlight the potential for endemic EEEV to emerge in Vermont and cause disease in the human population, especially given the established presence of EEEV in local wildlife [21,22]. Future studies examining the seroprevalence of EEEV in humans living in endemic areas will further elucidate the risk to human populations and aid development of preventative measures and targeted surveillance. Similarly, in depth vector prevalence and blood-meal identification studies will enhance our knowledge of likely transmission dynamics.
